# Spontaneous Type 2 Diabetic Rodent Models

**DOI:** 10.1155/2013/401723

**Published:** 2013-02-28

**Authors:** Yang-wei Wang, Guang-dong Sun, Jing Sun, Shu-jun Liu, Ji Wang, Xiao-hong Xu, Li-ning Miao

**Affiliations:** Department of Nephrology, Second Hospital of Jilin University, Changchun 130041, China

## Abstract

Diabetes mellitus, especially type 2 diabetes (T2DM), is one of the most common chronic diseases and continues to increase in numbers with large proportion of health care budget being used. Many animal models have been established in order to investigate the mechanisms and pathophysiologic progress of T2DM and find effective treatments for its complications. On the basis of their strains, features, advantages, and disadvantages, various types of animal models of T2DM can be divided into spontaneously diabetic models, artificially induced diabetic models, and transgenic/knockout diabetic models. Among these models, the spontaneous rodent models are used more frequently because many of them can closely describe the characteristic features of T2DM, especially obesity and insulin resistance. In this paper, we aim to investigate the current available spontaneous rodent models for T2DM with regard to their characteristic features, advantages, and disadvantages, and especially to describe appropriate selection and usefulness of different spontaneous rodent models in testing of various new antidiabetic drugs for the treatment of type 2 diabetes.

## 1. Introduction

In nearly all countries of the world, diabetes mellitus is one of the most common chronic diseases and continues to increase in numbers and significance, because of the reduced physical activity and increased obesity caused by changing lifestyles. It is estimated that the global figure of diabetes patients is about 250 million currently, and the number is expected to be 380 million by 2025 [1]. Between 2010 and 2030, there will be a 69% increase in number of adults with diabetes in developing countries and a 20% increase in developed countries [2]. In North America, about 90–95% of all cases of diabetes are type 2 diabetes mellitus (T2DM), and the population over 65 with T2DM is about 20% [3]. T2DM may result in severe complications, including renal failure, blindness, slow healing wounds, and arterial diseases [4]. About 5–10% of the total health care budget has been used for T2DM in many countries.

 In a general classification, diabetes mellitus is divided into two groups: type 1 diabetes mellitus (T1DM) and T2DM. T1DM or insulin dependent diabetes mellitus (IDDM) is an autoimmune disease that leads to destruction of islet beta cells in the pancreas, which results in a complete halt of insulin production [5], while T2DM, also called noninsulin dependent diabetes mellitus (NIDDM), is a heterogeneous disorder characterized by a progressive decline in insulin action (insulin resistance) in liver and peripheral tissues, accompanied by the inability of beta cells to compensate for insulin resistance (pancreatic beta cell dysfunction) leading to overt hyperglycemia [6]. Insulin resistance, characterized by reduced responsiveness to normal circulating concentrations of insulin, is a common feature of almost all patients with T2DM, and it plays a key role at the beginning and in the development of whole process of T2DM [7]. The presence of insulin resistance leads to increased beta cell insulin secretion with compensatory hyperinsulinemia [8]. Impaired function of beta cell will cause deterioration in glucose homeostasis, at this point, insulin secretion cannot keep pace with the underlying insulin resistance and glucose intolerance, then T2DM occurs [9]. In patients destined to develop T2DM, the beta cell compensatory response declines, and then develop relative or absolute insulin insufficiency [10]. 

Previous studies have demonstrated that T2DM is a multifactorial disease in which genetic factors consisting of multiple susceptibility genes and environmental factors contribute to the disease development, and the pathogenesis of T2DM is still unclear now [11]. Because of ethical considerations, clinical studies of human diabetes, especially disease pathology research is constrained to a certain extent, thus using various animal models can induce the disease by a different mechanism but with the similar or same results and offer promise of new insights into human diabetes, which is advantageous in biomedical studies owing to the uncertain etiology of T2DM and its causes are multifarious [4]. Rodent seems to be the most suitable model for the study of T2DM because of the small size, short generation interval, easy availability, and economic considerations [12].

In this study, we aim to review the current available spontaneous rodent models for T2DM with regard to their characteristic features, advantages, and disadvantages in order to investigate whether they can replicate the pathophysiological process of T2DM.

## 2. Spontaneous Type 2 Diabetic Rodent Models

Right now, there are lots of rodent models available for the study of T2DM, but some of them may not always be satisfactory to simulate human T2DM totally due to the large heterogeneity in the latter. Obviously, no single rodent model can represent the onset and development of human T2DM in all details. Taken together, the existing rodent models provide a rich array of opportunities for investigators to study the complex pathogenesis and pathophysiological process of T2DM [13]. There is no fully unified classification criteria for rodent models of T2DM, and in this paper various types of rodent models of T2DM will be divided into spontaneously diabetic rodent models, artificially induced diabetic rodent models, and transgenic/knockout diabetic rodent models on the basis of their strains, features, advantages, and disadvantages [14]. Among these large number of animal models of T2DM, the spontaneous type 2 diabetic rodent models are considered the most outstanding and most useful. We will respectively introduce several spontaneous rodent models of T2DM in order to investigate the process of insulin resistance of T2DM in human in the following part.

### 2.1. Spontaneous Type 2 Diabetic Obese Rodent Models

Obesity and the consequent insulin resistance are major features of T2DM in human beings, and they play a key role at the beginning and in the development of the whole process of T2DM [7]. Consequently, obese animal models of T2DM have been used to simulate the complex pathogenesis and pathophysiological process of human disease and to gain insights into the human condition. The *ob/ob* mouse, *db/db* mouse, and Zucker fatty (*fa/fa*) rat are the most typical examples of T2DM obese models with monogenic background. On the other hand, the KK mouse, the NZO mouse, the OLETF rat, and the NSY mouse are the representation of obesity-induced diabetes models with polygenic background [15]. Since these models show severe obesity, hyperinsulinemia, and insulin resistance throughout their lives, the agents like insulin sensitizers, antiobesity, and some other antihyperglycemia agents, which decrease the body weight and improve peripheral insulin sensitivity, have been largely tested by making use of these obese rodent models [16, 17].

#### 2.1.1. *ob/ob* Mouse

Leptin, the product of the *ob *gene, may be a partial factor contributing to insulin resistance [18]. The *ob/ob* mouse strain, from the Bar Harbor-Jackson laboratory, has a well-known feature of leptin deficiency because of the mutation identified in leptin gene leading to severe insulin resistance. The* ob* gene was transferred from the stock of origin onto the B/6 genomic background and was located on chromosome 6 [19]. In early 1970s, these *ob/ob* mice were used to investigate the pathogenesis of insulin resistance as the first rodent model. These *ob/ob *mice exhibit rapid gain in body weight and may reach three times of the normal weight of wild-type controls. In addition to obesity, early in the life of these mice, insulin resistance and hyperinsulinemia occur, which are out of proportion to their adiposity at early stages [20]. In the *ob/ob* model, hyperinsulinemia manifests at 3 to 4 weeks of age together with hyperphagia and insulin resistance. On the contrary, leptin treatment in *ob/ob *mice causes decrease of both glucose and insulin levels within hours of administration before either food intake or body weight changes [21]. Similarly, in normal rodents leptin also has a clear insulin-sensitizing effect acutely and after chronic administration [22]. In many rodent models of obesity, increased glucocorticoids also can mediate both the hyperphagia and insulin resistance due to leptin deficiency or resistance. In *ob/ob *mice, at least in rodents, suppressing hypothalamic-pituitary-adreno-cortical (HPA) axis by leptin replacement may be an important component of leptin action on insulin sensitivity [23].

The primary defect in the* ob/ob *mice is from neuroendocrine origin, but the exact aberration and its site are not yet fully known. Its expression is a lack of satiety control at the hypothalamic and/or pituitary level. The major action site of leptin is hypothalamus, where neurons are directly regulated by leptin reside [24]. Neuropeptide Y (NPY), produced by hypothalamus, is a neuromodulator implicated in the control of energy balance and in the hypothalamus of *ob/ob* mice. Overproduced as a central effector of leptin deficiency, NPY is associated with obesity, a typical symptom of T2DM and infertility, and eventually plays a major role on insulin action or secretion regulation in central nervous system (CNS) [25]. 

The symptom of T2DM of *ob/ob *mice attenuates with age, being manifested by the continuous decline of plasma insulin levels in the second year of life, with a consequent effect of glucose tolerance and insulin resistance improvement and a loss of adipose body weight. It is interesting to notice that *ob/ob* mice are not known to develop major diabetic complications despite the marked insulinemia in contrast to other species [26]. 

#### 2.1.2. *db/db* Mouse

The diabetes *db *gene mutation occurred spontaneously in the leptin-receptor-deficient C57BL/KsJ strain of mice and is originally derived from autosomal recessive mutation on chromosome 4 with complete penetrance, originating from Bar Harbor, Maine [27]. The *db/db* mouse can be considered as having a natural history that closely parallels to human beings. It becomes hyperphagic, hyperinsulinemic, and insulin resistant early in life (within 2 weeks of age), then develops obesity at the age of 3 to 4 weeks. The hyperglycemia becomes manifest at the age of 4 to 8 weeks due to beta cell failure. At this time, the insulin secretion of pancreatic beta cell depletes and the hyperinsulinemia recedes, the mouse exhibits ketosis, gradual body weight loss, and deceases no longer than 8 to 10 months [28]. In view of this, the sequence of events in this model appears to mimic human T2DM, but it seems not compatible to investigate the complication of T2DM because of the short life span. However, it is reported that the *db/db *mouse was used to investigate the renal and microvascular diabetic complications [29, 30].

Leptin controls food intake and neuroendocrine function by activating the long form of leptin receptor and ultimately regulates adiposity which influences insulin sensitivity. It also regulates glucose homeostasis independent on the energy balance in the same way [31]. Owing to alternative splicing, the leptin receptor which belongs to the cytokine receptor class I superfamily has several well-known isoforms in mouse, while the long form LEPR B isoform (LEPR-B) is the only STAT3 signaling-competent isoform [32]. Compared with *ob/ob *mouse, the *db/db* mouse represents similar hypothalamic disturbances and NPY abnormalities as a result of lack of leptin receptors. At the same time, the leptin is still produced but it cannot activate the leptin receptors and restrain obesity and hyperinsulinemia induced by NPY. In order to manifest this viewpoint, some investigators treated *db/db* mouse with exogenous leptin, the results showed that leptin administration has no effect on food intake and body weight gaining [21].

#### 2.1.3. KK (Kuo Kondo) Mouse

The KK mouse originating from Japan is a polygenic model of obesity and T2DM. It was crossed with the Bar Harbor C57BL/6J mouse, and the body size of original strain of this mouse was large [33]. In 1957, Kondo et al. reported the KK mouse at first, which spontaneously exhibited distinct adiposity, hyperglycemia, and hyperinsulinemia [34]. At about 2 months of age, the KK mouse manifested moderate obesity due to hyperphagic, which was associated with insulin resistance, compensatory hyperinsulinemia, and islet cell hyperplasia. The insulin resistance and hyperinsulinemia reached to the peak at about 5 months and returned to normal at 9 to 12 months due to beta cell failure [35]. 

The KK mouse bred several substrains throughout the world, and they vary both genetically and phenotypically from each other. Among these substrains, KK/Ay mouse, also named as Yellow KK mouse, developed as a result of the dominant mutation of yellow agouti (Ay) gene in Japanese KK [36]. Homozygous KK/Ay mouse will die before implantation or shortly thereafter, whereas heterozygous KK/Ay mouse can grow up representing severe obesity, insulin resistance, hyperglycemia, and hyperinsulinemia at the age of about 8 weeks [37]. The hyperglycemia and hyperinsulinemia of KK/Ay mouse continue to develop along with the age and it has no need to maintain a high energy diet in order to become diabetic. For the past many years, the KK/Ay mouse was used as mild hyperglycemia model for studying the treatment to prevention and intervention with obesity.

#### 2.1.4. Zucker Fatty (*fa/fa*) Rat

Zucker fatty (*fa/fa*) rat, along with finding of the spontaneous mutation “obese” (fatty) in the rat stock of Sherman and Merck, was found by Zucker in Harriet Bird Memorial Laboratory during 1974 and 1975. On chromosome 5 of the Zucker fatty (*fa/fa*) rat, the simple autosomal recessive (*fa*) gene is mutant spontaneously, which results in hyperphagia and early onset of obesity. At the age of 4 weeks, the Zucker fatty (*fa/fa*) rat from the obese strain gained weight more rapidly because of the increased growth of subcutaneous fat depot, and it had a considerably higher body weight at about the age of 9 weeks [38, 39]. Like the *db/db* mouse model, the hyperphagia and obesity in the Zucker fatty (*fa/fa*) rat are attributed to hypothalamic defect in leptin receptor signaling, which is also associated with mild hyperglycemia, insulin resistance, mild glucose intolerance, hyperlipidemia, hyperinsulinemia, and moderate hypertension [19].

#### 2.1.5. Zucker Diabetic Fatty (ZDF) Rat

The Zucker diabetic fatty (ZDF) rat originated from an outbred strain. The Zucker diabetic fatty (ZDF) rat was an inbred line of Zucker fatty rat developing into a genetic model, which was established in 1985 and in 1991. Compared with Zucker fatty rats, male ZDF rats become less obese but more insulin resistant, and then rapidly progress to frank diabetes because of the lack of sufficient insulin secretion required adequate compensation for the insulin resistance [40]. The male ZDF rat represents hyperglycemia at about 7 weeks of age, and fully diabetic at 12 weeks. The serum insulin levels of male ZDF rat reach the peak at about 7 to 10 weeks, but as soon as the beta cells of pancreas cannot respond to glucose stimulus, the insulin levels drop subsequently as pancreatic cease to [41]. The female ZDF rat develops diabetes just on a diabetogenic diet unlike male ZDF rat.

It has been reported that in the progression of ZDF rat, the decrease of beta cell glucose transporter 2 (GLUT-2) membrane receptors and the concomitant loss of muscle glucose transporter 4 (GLUT-4) transporters are responsible for the impaired insulin secretion and subsequent hyperglycemia. The activity of GLUT-4 receptors decreased in adipose tissue and skeletal muscles of ZDF rat, which leads to the decreased beta cell transport ability together with the peripheral insulin resistance [42]. The decrease of GLUT-2 membrane receptors coupled with insulin resistance and beta cell dysfunction in ZDF rat can be commonly used to simulate the mechanism of human T2DM and test insulin sensitizers and other various agents.

#### 2.1.6. New Zealand Obese (NZO) Mouse

The New Zealand strain of obese mice, a model of polygenic obesity, which was obtained by selective inbreeding from a stock colony in the Hugh Adam Department of Cancer, considerably gains weight during the first 10 weeks of life as a result of hyperphagia and reachs to peak at about 12 months, coupled with the corresponding hyperglycemia and hyperinsulinemia [43]. Unlike other obese models, the NZO mouse manifests insulin resistance at an early age and gradually represents hyperleptinemia together with leptin resistance. With the growth of NZO mouse, hyperglycemia and impaired glucose tolerance increase continuously, and the level of blood glucose reaches 300–400 mg/dL at the age of 20 to 24 weeks [44]. In NZO mouse, the hyperglycemia occupies a leading position despite the fact that the level of insulin is lower than that in other obese models because of the distinct peripheral insulin resistance and increased gluconeogenesis [45]. Although NZO mouse is a rare preferred model, it becomes a useful model for studying the relationship between autoimmunity, obesity, and diabetes [46], and its new recombinant congenic strains are used to study the “diabesity” and its treatment [47].

#### 2.1.7. Otsuka Long-Evans Tokushima Fatty (OLETF) Rat

The Otsuka Long-Evans Tokushima Fatty (OLETF) rat was obtained by selectively inbreeding from an outbred colony of Long-Evans rats purchased from Charles River, Canada, develops mild to moderate obesity early at about 4 weeks with hyperglycemia later in life at around 18 to 25 weeks age [48]. OLETF rats exhibit the characteristics of innate polyphagia, mild obesity, late onset of hyperglycemia, hyperinsulinemia, hypertriglyceridemia, hypercholesterolemia, chronic course disease, and clinical onset of diabetes mostly in males, which are similar to the pathophysiologic progress of human T2DM [49]. Many recessive genes on several chromosomes including the X chromosome are involved in the induction of diabetes in OLETF rats. It is also observed that the OLETF rats carry a null allele for the cholecystokinin A receptor which may be related to the regulation of food intake [50, 51]. The pancreatic islets of OLETF rats change progressively, and less than 9 weeks of age, the islets just exhibit mild lymphocyte infiltration, then show hyperplastic alterations and fibrosis in or around islets at around 10 to 40 weeks of age, finally more than 40 weeks, represent atrophy of islets [52]. With OLETF rats, many investigators carried out some researches to test exogenous insulin [53] and antidiabetic drugs such as Cilnidipine [54], Acarbose [55], Pioglitazone [56], and so on. 

#### 2.1.8. Nagoya-Shibata-Yasuda (NSY) Mouse

The Nagoya-Shibata-Yasuda (NSY) mouse, an inbred polygenic animal model of T2DM, was established by selective inbreeding from the Jc1: ICR mouse, from which the NOD mouse was also derived [57]. The NSY mouse closely imitates human T2DM in which the characteristics of NSY mouse are mild obesity with abdominal and visceral fat accumulation, accompanied by impaired insulin secretion and moderate insulin resistance contributing to diabetes development in an age-dependent manner [57]. For NSY mice, there is a marked gender difference that almost all males develop diabetes, while the percentage for females is only about 30% [57]. The NSY mouse is also a polygenic model of T2DM, and three major susceptibility loci (*Nidd*1*n*, *Nidd*2*n*,and *Nidd*3*n*) have been mapped on chromosomes 11, 14, and 6 [58]. The NSY mouse is particularly useful for studying the age-related damages and phenotypes of T2DM, as well as the undiscovered genetic differentiations and correlations between T1DM and T2DM.

#### 2.1.9. Tsumura Suzuki Obese Diabetes (TSOD) Mouse

In 1992, the Tsumura Suzuki Obese Diabetes (TSOD) mouse, accompanied with the other strain named Tsumara Suzuki Non-Obese (TSNO), was established by Tsumura and Suzuki through repeatedly selective inbreeding of obese male mice of ddY strain [59]. Differing from the TSNO mouse that does not become obese, the TSOD mouse exhibits polygenic obesity and insulin resistant at about 2 months old, which results in hyperinsulinemia and hyperglycemia, but just in males [60]. In TSOD mouse, pancreatic islets are hypertrophic and **β**-cell mass increases, which controls the blood glucose levels so that the severe diabetes does not develop [61]. The impaired GLUT4 translocation in both skeletal muscle and adipocytes of TSOD mouse is one of the important reasons for the reduced insulin sensitivity and insulin resistance [62]. It is investigated that many susceptibility loci have been mapped on chromosomes 11, 1, and 2, which are closely related to obesity, hyperglycemia, and hyperinsulinemia of TSOD mouse, and the combination of these genetic loci may lead to the symptoms of TSOD mouse similar to human T2DM [63].

#### 2.1.10. M16 Mouse

M16 mice, a unique line of mice created through election for 3 to 6 weeks weight gain for many generations from an outbred ICR base stock in Institute of Cancer Research in London, are a new animal model to simulate human obesity and T2DM [64]. Compared with ICR, both males and females of M16 mice gain weight at early age and maintain moderate obesity at all ages because of hyperphagia, accompanied by increased body fat percentage, fat cell size and numbers, and organ weights [65]. At 8 weeks of age, all M16 mice exhibit hyperglycemic, hyperinsulinemic, and hypercholesterolemic relative to ICR, but the fasted blood glucose levels are, respectively, 56 percent higher in males and 22 percent higher in females [66].

#### 2.1.11. Spontaneously Hypertensive Obese (SHR/Ncp) Rat

The spontaneously hypertensive obese rat (SHR/Ncp), an obese model of T2DM with hypertension, is derived by mating Koletzky rats which are obese with hypertensive SHR rats [67]. Part of the resultant descendants exhibits obesity with normotensive or mildly hypertensive, which is determined by an autosomal recessive trait [67]. The characteristics of SHR/Ncp rats are hyperphagia and early onset of obesity, insulin resistance, impaired glucose tolerance, hyperinsulinemic, and normal or slight hyperglycemia [12].

#### 2.1.12. Spontaneously Diabetic Torii (SDT) Fatty (*fa/fa*) Rat

A new model of obese type 2 diabetes, spontaneously Diabetic Torii (SDT) fatty (*fa/fa*) rat, was established by introducing the *fa* allele of the Zucker fatty rat into the spontaneously Diabetic Torii (SDT) rat genome via the Speed Congenic Method [68]. Compared to male SDT rats, SDT fatty (*fa/fa*) rats showed overt obesity, and hyperglycemia and hyperlipidemia at younger ages (5~6 weeks), which is associated with hyperphagia by an induced disorder of leptin action, and plasma triglyceride (TG) and total cholesterol (TC) levels in SDT fatty (*fa/fa*) rats were significantly higher than those in original SDT rats. Furthermore, with an early incidence of diabetes mellitus, diabetes-associated complications (such as renal lesions and cataract) in the SDT fatty (*fa/fa*) rat are seen at younger ages than in the SDT rats [69–71]. The pharmacological effects of antidiabetic drugs, such as metformin, pioglitazone, and dipeptidyl peptidase-4 inhibitor, have been tested on SDT fatty (*fa/fa*) rats [72]. SDT fatty rat is expected to be a useful model for analysis of diabetic complications and the evaluation of drugs used for metabolic disease.

### 2.2. Spontaneous Type 2 Diabetic Nonobese Rodent Models

It is demonstrated that human T2DM can also exist in the absence of obesity. Thus, it is necessary for developing nonobese models to study this condition of the T2DM. The GK (Goto-Kakizaki) rat, Cohen diabetic rat, and Spontaneously Diabetic Torii (SDT) rat are the typical examples of nonobese models of T2DM. These nonobese rodent models are very useful for studying the mechanisms of diabetes complications (e.g., renal, retinal, and peripheral nerves lesions) [15]. However, because of their absence of obesity and hypoinsulinemia, only very few studies on drug testing using these models have been reported in the literature [73]

#### 2.2.1. Goto Kakizaki (GK) Rat

In 1973, Goto and his collaborators in Japan developed the Goto Kakizaki (GK) rat, a polygenic nonobese model of T2DM with early and relatively stable hyperglycemia, hyperinsulinemia, and insulin resistance, through selective inbreeding of nondiabetic Wistar rats with mild glucose intolerance over many generations [74]. For GK rats, because of the reduced number of islets at birth, both insulin resistance and impaired insulin secretion are present at their adult lives, coupled with moderate but stable fasting hyperglycemia which is present at the end of the first 2 weeks. And after 8 weeks, hyperglycemia degenerates and insulin secretion of the islets stimulated by glucose is more severely impaired, but generally, during its lifetime, fasting glucose remains mild and stable and rises only after challenge with glucose [75]. For GK rats, 60% of pancreatic *β*-cell mass decreased along with the distinct defects on *β*-cell function, because of the secondary loss of *β*-cell differentiation manifested by the three genetic loci correlated with impaired insulin secretion and glucotoxicity [75]. In the pancreatic islets of GK rats at 2 months of age, the deposition of fibrous and nonamyloid material bring about the result that the islets become transmuted in shape but do not increase in total area. With the exception of the defects of pancreatic *β*-cell mass, the decrease of insulin sensitivity in peripheral tissues and hepatic glucose overproduction are also mechanisms of development of hyperglycemia and insulin resistance [76]. For GK rat, developing some features of complications of diabetes can be used to study the diabetic complications seen in humans, including glomerulopathy [49], peripheral neuropathy [77], and retinopathy [78]. The GK rat is one of the useful animal models for studying the defects of pancreatic *β*-cell mass and pathophysiologic progress of T2DM and its complications. 

#### 2.2.2. Cohen Diabetic Rat

Cohen diabetic rat, an exceptional genetical model derived from diet-induced T2DM model by placing the rat on a synthetic 72% sucrose-copper-poor diet for 2 months, displays many features of the human T2DM [79]. These features include nonobesity, hyperinsulinemia, and insulin resistance. Meanwhile, the Cohen diabetic rat expresses genetic susceptibility (sensitivity and resistance) to a carbohydrate-rich diet, a central feature of T2DM in human beings. Nevertheless, the Cohen diabetic rat has many shortcomings, a major of which is that it has never been systematically characterized in terms of phenotype or genotype. For this reason, the Cohen diabetic rat model has been studied until recently only to a limited extent after being established nearly 30 years ago [80].

#### 2.2.3. Spontaneously Diabetic Torii (SDT) Rat

Spontaneously Diabetic Torii (SDT) rat is a new spontaneously nonobese diabetic strain derived from the Sprague-Dawley rat in 1997 at Torii Pharmaceutical Co., Ltd. (Tokyo, Japan) [81]. It has been found that the distinct characteristics of this model are glucose intolerance, hyperglycemia, hypoinsulinemia, and hypertriglyceridemia. It was reported that there is a major cumulative incidence of diabetes in male rats (100% by 40 weeks of age) than in female rats (33.3% even by 65 weeks of age) [82]. Because of the chronic severe hyperglycemia, SDT rats develop diabetic retinopathy (DR) [82–84], diabetic peripheral neuropathy [85, 86], and diabetic nephropathy [87]. This rodent model is suitable for investigating the complications of human T2DM [88].

## 3. Conclusion

With worldwide rises of T2DM incidences, many of the animal models have been established by different mechanisms to describe similar characteristic features of this human disease and new therapeutic methods still need to be found. Among these large numbers of animal models of T2DM, the spontaneous rodent models are the most outstanding, distinctive, and useful one, because of the small size, short generation interval, easy availability and economic considerations. Additionally, many of the spontaneous animal models can commendably simulate the characteristics of T2DM, such as obesity, insulin resistance, hyperinsulinemia, hyperglycemia, and hyperlipemia. However, every spontaneous rodent model of T2DM has either advantages or disadvantages and each of them cannot replicate all characteristic features of T2DM because of the complex, heterogeneous, multifactorial syndrome of this disease. So, in the future, more promising animal models that closely simulate human T2DM regarding all aspects of T2DM are needed urgently.
